# Supplementary material for: Enumerating Circulating Tumor Cells with a Self-Assembled Cell Array (SACA) Chip: A Feasibility Study in Patients with Colorectal Cancer
Source: Cancers (Basel). 2019 Jan 8;11(1):56. doi: 10.3390/cancers11010056 (PMC6356678; doi:10.3390/cancers11010056)
Supplement: Supplementary file 1 [file cancers-11-00056-s001.pdf]

Supplementary Materials: Enumerating circulating tumor cells with self-assembled cell array (SACA) chip: a feasibility study in patients with colorectal cancer

Hsueh-Yao Chu, Long-Sheng Lu, Wanying Cho, Shin Yao Wu, Yu-Cheng Chang, Chien-Ping Lin, Chih-Yung Yang, Chi-Hung Lin, Jeng-Kai Jiang and Fan-Gang Tseng

The performance of SACA chip to detect rare cells was measured. HeLa cells were diluted inwhite blood cells at indicated proportions from 1:10,000 to 1:10,000,000. The preparation was submitted to SACA chip and stained with anti-CD44 (green) and anti-CD45 (red). At 1:10,000,000 dilution, 15.2% HeLa cells were lost during preparation (left panel), and single target HeLa cell was easily identified under fluorescence microscope.

HeLa: WBC	Mixed HeLa cells number	Detected HeLa cells number	Average deviation value
1:10,000	29<br>34<br>41	27<br>30<br>38	8%
1:100,000	35<br>39<br>42	33<br>34<br>38	9%
1:1,000,000	34<br>36<br>39	30<br>31<br>34	12.7%
1:10,000,000	33<br>37<br>38	29<br>30<br>32	15.2%

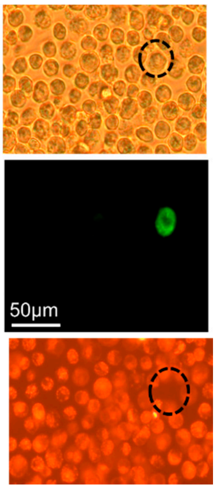

Figure S1. Rare cell detection from human blood by SACA chip.

CTC clusters were detected in blood samples from CRC patients. Representative images from one case were shown in upper panel in Figure S2. CTC clusters were present in PB of 7 non-metastatic CRC patients, (mean ± SEM, 2.85 ± 0.63) and IMV (mean ± SEM, 4.30 ± 1.45) of 3 non-metastatic CRC (lower panel, Figure S2). The difference of CTC cluster numbers between PB and IMV was not statistically significant.

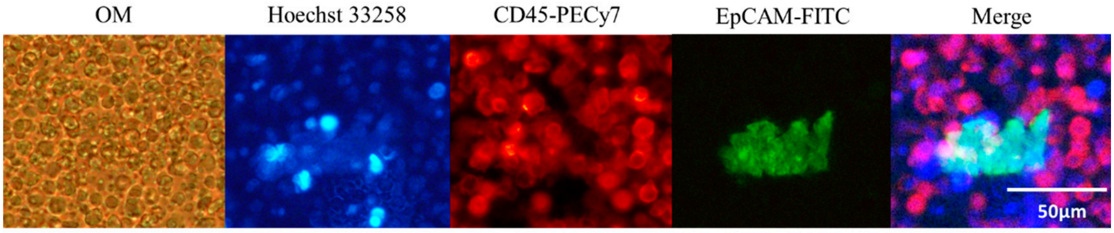

Figure S2. Images of CTC clusters from CRC patients. The images were acquired on the microscope at 10× magnification. OM: bright field images under optical microscopy.

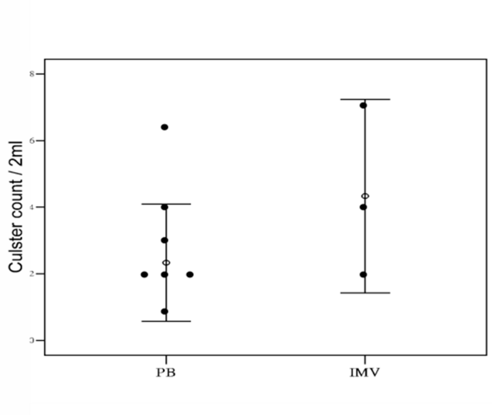

Figure S3. Detection of CTC clusters from CRC patients.
